# Supplementary material for: Plasma Oxalate as a Predictor of Kidney Function Decline in a Primary Hyperoxaluria Cohort
Source: Int J Mol Sci. 2020 May 20;21(10):3608. doi: 10.3390/ijms21103608 (PMC7279271; doi:10.3390/ijms21103608)
Supplement: Supplementary file 1 [file ijms-21-03608-s001.pdf]

Supplemental Table S1. ESKD rates per plasma oxalate quartile during follow-up, by CKD stage

	CKD Stage			
	Stage 1 (≥90)	Stage 2 (60-<90)	Stage 3a (45-<60)	Stage 3b (30-<45)
**Number f/u intervals beginning at this level**				
Q1	48	75	42	10
Q2	41	69	41	9
Q3	40	74	41	10
Q4	42	70	41	9
**Person-years at this level**				
Q1	117	191	84	3
Q2	81	200	57	1
Q3	89	125	64	4
Q4	73	108	42	7
**ESRD events at this level**				
Q1	0	1	2	13
Q2	1	3	3	12
Q3	1	3	2	9
Q4	2	8	12	3
**ESRD rate per 100 person years (95% CI)**				
Q1	0.0 (0.0, 3.2)	0.5 (0.0, 2.9)	2.4 (0.3, 8.6)	23.1 (4.8, 67.4)
Q2	1.2 (0.0, 6.9)	1.5 (0.3, 4.4)	5.3 (1.1, 15.4)	8.3 (0.2, 46.4)
Q3	1.1 (0.0, 6.3)	2.4 (0.5, 7.0)	3.1 (0.4, 11.3)	44.4 (12.1, 114)
Q4	2.7 (0.3, 9.9)	7.4 (3.2, 14.6)	28.6 (14.8, 49.9)	233 (93.8, 481)
